# Sustainment of Hydroxyurea Adherence in Patients With Sickle Cell Disease

**DOI:** 10.1001/jamanetworkopen.2026.11257

**Published:** 2026-05-06

**Authors:** Andrew M. Heitzer, Zachary Wooten, Guangjin Luo, Marsha Treadwell, Allison A. King, Victor R. Gordeuk, Nirmish Shah, Christina M. Abrams, Sarah McCuskee, Siera Gollan, Jennifer Longoria, Jane S. Hankins

**Affiliations:** 1 Department of Psychology and Biobehavioral Sciences, St Jude Children’s Research Hospital, Memphis, Tennessee; 2 Department of Biostatistics, St Jude Children’s Research Hospital, Memphis, Tennessee; 3 Department of Pediatrics, Division of Hematology, University of California San Francisco, Oakland; 4 Department of Pediatrics, Washington University in St Louis, St Louis, Missouri; 5 Department of Medicine, Washington University in St Louis, St Louis, Missouri; 6 Department of Medicine, University of Illinois at Chicago, Chicago; 7 Department of Medicine, Duke University, Durham, North Carolina; 8 Department of Pediatrics, Medical University of South Carolina, Charleston; 9 Department of Emergency Medicine, Icahn School of Medicine at Mount Sinai, New York, New York; 10 Center for Blood Disorders, Augusta University, Augusta, Georgia; 11 Department of Hematology, St Jude Children’s Research Hospital, Memphis, Tennessee; 12 Department of Global Pediatric Medicine, St Jude Children’s Research Hospital, Memphis, Tennessee

## Abstract

**Importance:**

Despite established treatment guidelines and strong therapeutic benefit, hydroxyurea remains underused among patients with sickle cell disease.

**Objective:**

To assess the change in self-reported hydroxyurea use (ie, taking the medication at all) and adherence (ie, the number of days taken per week) over time and to examine factors associated with the trajectory of hydroxyurea use and adherence.

**Design, Setting, and Participants:**

Data were collected through the Sickle Cell Disease Implementation Consortium, a longitudinal, multicenter, observational cohort study that recruited patients from 8 centers across the US. Data collection occurred from 2017 to 2022, and analyses were performed from January to October 2025. Data were collected via self-report survey at baseline and 3 follow-up surveys distributed yearly. Patients with sickle cell disease of all genotypes between the ages of 15 and 45 years were included.

**Exposure:**

Sickle cell disease.

**Main Outcomes and Measures:**

Hydroxyurea use was analyzed as a binary variable according to whether the participant was currently taking hydroxyurea. Among those taking hydroxyurea, adherence was analyzed as a continuous variable according to the number of days hydroxyurea was taken within the past week. Continuous variables were *z *scored to ensure model convergence, and odds ratios (ORs) are reported.

**Results:**

Of 2514 eligible participants, surveys were completed by 2207 participants at baseline, 1802 at follow-up 1, 1281 at follow-up 2, and 783 at follow-up 3. Of 2207 participants at baseline, 1265 (57.3%) were female, with a mean (SD) age of 28.06 (7.86) years. Overall rates of hydroxyurea use remained stable throughout the study (1089 of 2207 patients [49.3%] at baseline, 887 of 1802 patients [48.7%] at first follow-up, 609 of 1281 patients [47.5%] at second follow-up, and 378 of 783 patients [48.3%] at third follow-up). However, patients with HbSS/SB^0^-thalassemia showed declining use (790 of 1550 patients [50.9%] at baseline, 627 of 1276 patients [49.1%] at first follow-up, 429 of 905 patients [47.4%] at second follow-up, and 282 of 586 patients [48.1%] at third follow-up), whereas patients with other genotypes showed increasing use (299 of 657 patients [45.5%] at baseline, 250 of 526 patients [47.5%] at first follow-up, 168 of 352 patients [47.7%] at second follow-up, and 90 of 186 patients [48.4%] at third follow-up) across time points (OR, 0.76; 95% CI, 0.63 to 0.91; *P* = .004). Hydroxyurea adherence declined over time (−0.19 days/week/year; 95% CI, −0.24 to −0.14 days/week/year; *P* < .001). Executive difficulties were associated with worse adherence across time points (−0.17 days/week/year; 95% CI, −0.26 to −0.08 days/week/year; *P* < .001).

**Conclusions and Relevance:**

In this cohort study of patients with sickle cell disease, declining use of hydroxyurea among patients with HbSS/SB^0^-thalassemia genotypes raises important concerns about ongoing disease management. Adherence to hydroxyurea is negatively associated with cognitive factors that may be addressed through interventions targeting self-monitoring and/or behavioral activation.

## Key Points

**Question:**

Do hydroxyurea use (ie, taking the medication at all) and adherence (ie, days taken per week) change over time, and are they associated with patient characteristics among individuals with sickle cell disease?

**Findings:**

This cohort study of 2207 participants revealed that overall rates of hydroxyurea use remained stable (range, 47.5% to 49.3%) over 3 yearly follow-up surveys, but use was lowest among older participants. Hydroxyurea adherence significantly declined on average and was negatively associated with difficulties in executive functioning.

**Meaning:**

These findings suggest that hydroxyurea use and adherence decline after adolescence and that interventions to promote sustainment of hydroxyurea adherence are needed.

## Teaser

This cohort study assesses changes in self-reported hydroxyurea use and adherence over time and examines factors associated with the trajectory of hydroxyurea use and adherence among patients with sickle cell disease.

## Introduction

Sickle cell disease (SCD) is a monogenic blood disorder that causes multisystemic and progressive organ damage and affects approximately 100 000 individuals in the US.^1^ The primary disease-modifying treatment for SCD is hydroxyurea.^2,3,4^ Despite established treatment guidelines and strong evidence for its therapeutic benefit, hydroxyurea remains underused within the US. Claims data from multiple states suggest that less than one-half of patients with HbSS/HbSb^0^-thalassemia currently take the medication at all (ie, use),^5,6^ in part because of low prescription rates.^7^ Among users, less than half display adequate adherence.^8,9,10^

The World Health Organization introduced a framework of 5 factors that contribute to medication adherence, including health care system, medication, socioeconomic, condition, and patient factors.^11^ At the level of the health care system and medical practitioners, documented barriers include patient-practitioner trust,^12^ hydroxyurea supply and cost,^13^ and practitioner knowledge.^13^ Patients and practitioners have reported adverse effects and lack of perceived efficacy as medication-related barriers.^13,14^ Less is known about the influence of patient-related (eg, age), socioeconomic (eg, education), and condition-related (eg, pain) factors. Furthermore, most adherence barriers and adherence-promoting interventions have been studied in pediatric rather than adult patients with SCD.

Both use and adherence appear best in childhood, potentially because of parental support.^15,16^ For adolescents and young adults, some studies indicate better hydroxyurea use and adherence among young adults than among adolescents,^17^ whereas others show the opposite.^9^ Across the lifespan, older age is associated with lower hydroxyurea adherence,^18^ but it is unclear when or why these age-related changes occur.

Nonadherence to hydroxyurea is associated with multiple negative outcomes, including greater rates of hospitalizations and vaso-occlusive events, along with higher overall health care costs.^10^ Patients who do not use or are not adherent to hydroxyurea report worse health-related quality of life (including pain)^14,19^ and greater rates of depression.^20^ Hydroxyurea adherence may protect the brain from cerebrovascular insults^21,22^ and subsequent neurocognitive deficits.^23^ However, associations between adherence and mental health or cognitive functioning are based on cross-sectional studies.

Our first objective was to assess the change in self-reported hydroxyurea use and adherence over a 4-year period. Second, we examined whether condition-related (eg, genotype or hemoglobin), socioeconomic (education), and patient-related (eg, age) factors modified the trajectory of hydroxyurea use or adherence. Finally, we assessed associations between pain, depression, and executive deficits with hydroxyurea adherence using cross-lagged models to explore bidirectional longitudinal relationships. We hypothesized that hydroxyurea use and adherence would decline over the study period and that young adults (ages 18-24 years) would show the greatest decline. We estimated that self-reported depression and executive difficulties would be associated with worse future hydroxyurea adherence.

## Methods

### Procedures and Participants

The Sickle Cell Disease Implementation Consortium (SCDIC) is a multisite consortium in the US and includes 8 academic medical centers and 1 data coordinating center.^24^ Established in 2016, the SCDIC prospectively enrolled participants with SCD into a registry.^25^ Institutional review board approval was obtained at each SCDIC site, and written informed consent was obtained from each participant or their legal guardian before study enrollment. Results reporting adhered to Strengthening the Reporting of Observational Studies in Epidemiology (STROBE) reporting guideline.

Inclusion criteria for enrollment were as follows: age 15 to 45 years, confirmed diagnosis of SCD (subtypes HbSS, HbSC, HbSS/SB^0^-thalassemia, HbSO, HbSD, HbSE, or HbSF) by hemoglobin fractionation tests, and literacy in English. Participants were excluded if they had received a clinically successful bone marrow transplant. The recruitment of participants began in October 2017 through various venues at the discretion of each participating center.^26^ All data were collected between 2017 and 2022, and analyses were performed between January and October 2025.

Consented participants completed an enrollment survey and had baseline data on their disease characteristics abstracted from their medical records. The SCDIC registry contains 2514 participants with baseline results. Participants were excluded from this study if they did not complete items measuring executive difficulties, pain, and depression at baseline and follow-up 1. At each time point, if a participant did not report whether they used hydroxyurea, they were excluded (307 participants). The first follow-up survey was completed a mean (SD) of 1.33 (0.38) years after the baseline, the second was completed a mean (SD) of 2.44 (0.45) years after the baseline, and the third was completed a mean (SD) of 3.40 (0.52) years after the baseline.

### Outcome Measures

#### Demographic and Clinical Information

The SCDIC enrollment survey was developed by the SCDIC registry committee, which consisted of at least 1 SCD expert from each of the 8 participating sites.^27^ The survey assessed demographic, disease, and treatment variables as described elsewhere.^27^ Race and ethnicity (Asian, Black, Native American, White, and any other race not otherwise specified) were collected through a self-report survey completed online or over the telephone and are included here to describe the demographics of the patients.^27^ Information about the 8 participating sites and the participants at each site were described previously.^26^

On the baseline and follow-up surveys, patients were asked, “Are you currently taking hydroxyurea?” and “How many days did you take hydroxyurea in the past week?”^25^ Hydroxyurea use was analyzed as a binary variable according to whether they were currently taking hydroxyurea. Hydroxyurea adherence was analyzed as a continuous variable according to the number of days the medication was taken within the past week.^28^

#### Patient-Reported Outcomes

All patient-reported outcomes used by SCDIC were previously described in detail.^27,29,30^ In brief, surveys included items from several National Institutes of Health–developed HealthMeasures,^31^ including the Quality of Life in Neurological Disorders,^32^ Adult Sickle Cell Quality of Life Measurement Information System,^33^ and Patient-Reported Outcomes Measurement Information System.^34^

Items from the Quality of Life in Neurological Disorders Item Bank version 2.0, Cognitive Function Short-Form were used to assess cognitive symptoms. Consistent with previous work,^29^ 5 items were used to assess executive functioning. Four items from the Patient-Reported Outcomes Measurement Information System Short Form version 1.0 Depression 4a evaluated the frequency of depressive symptoms in the last week. At baseline, pain impact over the past 6 months was measured with 2 items from the Adult Sickle Cell Quality of Life Measurement Information System. At each follow-up, the same pain impact questions were asked, but with a 1-week rather than 6-month time window. Raw item responses for each measure were transformed to *z* scores (mean = 0; SD = 1) for analyses.

### Statistical Analysis

Descriptive analyses were conducted to characterize the patient cohort at each time point, using counts and percentages for categorical variables and the mean and SD for continuous variables. Baseline characteristics were compared between participants reporting hydroxyurea use and those not using hydroxyurea. The baseline categorical variables were compared using χ^2^ tests, and the continuous variables were compared using a 2-sided Wilcoxon rank-sum test. The data were summarized across the 4 time points in R statistical software version 4.4.0 (R Project for Statistical Computing).^35^ *P* < .05 was considered statistically significant.

To evaluate the variables of interest that affected hydroxyurea use, the lmer and lme4 packages were used to fit a linear mixed-effects (LME) model with a logit link function for the binary outcome variable of hydroxyurea use and random intercepts for the patients.^36,37^ Time was parameterized as a continuous variable of years since baseline. Furthermore, we evaluated the time interaction terms between time and age group, sex, education, hemoglobin, executive function, depression, and pain to determine whether the association between those variables and hydroxyurea use differed over time. The continuous variables were *z* scored to ensure model convergence, and the odds ratios (ORs) are reported. For the cohort of patients reporting hydroxyurea use, LME models were used to examine the days of hydroxyurea used in the last week, with the same covariate and interaction structure as previously described.

Finally, the bidirectional longitudinal associations between the days of hydroxyurea taken in the last week and executive function, depression, and pain were examined using a cross-lagged panel model (CLPM) with the lavaan package.^38^ We chose to use CLPMs because our goal was to evaluate any possible reciprocal lagged associations between symptoms and hydroxyurea adherence across successive time points, while being able to account for previous time point associations through the stability paths. Separate models were estimated for the association of days of hydroxyurea taken with executive difficulties, depression, and pain. Each CLPM included stability estimates and cross-lagged paths across time points with time-invariant baseline variables for age group, sex, education, genotype, and hemoglobin included as confounders. Here, the cross-lagged estimates are interpreted as bidirectional associations that can reflect both within-person changes and between-person changes.

## Results

The final analytic sample included 2207 participants at baseline, 1802 at follow-up 1, 1281 at follow-up 2, and 783 at follow-up 3. Table 1 shows the demographic, treatment, and medical characteristics of 1089 participants taking hydroxyurea and 1118 participants not taking hydroxyurea at baseline. Of the 2207 participants at baseline, 1265 (57.3%) were female, with a mean (SD) age of 28.06 (7.86) years. Those taking hydroxyurea reported taking the medication a mean (SD) of 6.53 (2.3) days per week at baseline. eTables 1 and 2 in Supplement 1 show descriptive summaries of demographic and other clinical characteristics at baseline and at each follow-up time point, for the overall cohort and the among participants reporting hydroxyurea use.

**Table 1.** Baseline Demographic and Medical Characteristics of the Study Participants

Characteristic	Participants, No. (%) (N = 2207)		*P* value^a^
	Hydroxyurea use (n = 1089)	No hydroxyurea use (n = 1118)	
Sex			
Female	565 (51.9)	700 (62.6)	<.001
Male	524 (48.1)	418 (37.4)	
Race			
Asian	1 (0.1)	3 (0.3)	.20
Black	1066 (97.9)	1098 (98.2)	
Native American	0	3 (0.3)	
White	5 (0.5)	3 (0.3)	
Other^b^	17 (1.6)	11 (1.0)	
Age group, y			
15-17	109 (10.0)	80 (7.2)	<.001
18-24	340 (31.2)	280 (25.0)	
25-34	410 (37.6)	473 (42.3)	
35-45	227 (20.8)	282 (25.2)	
Unknown	0	0	
Missing	3 (0.3)	3 (0.3)	
Annual household income, $			
≤25 000	512 (47.0)	532 (47.6)	.72
25 001-50 000	208 (19.1)	232 (20.8)	
50 001-75 000	102 (9.4)	110 (9.8)	
75 001-100 000	60 (5.5)	53 (4.7)	
≥100 001	73 (6.7)	71 (6.4)	
Unknown	134 (12.3)	120 (10.7)	
Education			
Less than high school	34 (3.1)	26 (2.3)	.89
Some high school	154 (14.1)	150 (13.4)	
High school graduate or General Educational Development test	297 (27.3)	308 (27.5)	
Some college or vocational training	336 (30.9)	366 (32.7)	
College graduate	156 (14.3)	155 (13.9)	
Some graduate or professional school	21 (1.9)	21 (1.9)	
Graduate or professional degree	72 (6.6)	79 (7.1)	
Missing	19 (1.7)	13 (1.2)	
Marital status			
Not applicable (child)	132 (12.1)	109 (9.7)	<.001
Married	104 (9.6)	129 (11.5)	
Living as married	23 (2.1)	53 (4.7)	
Divorced or separated	52 (4.8)	74 (6.6)	
Widowed	0	0	
Never married	765 (70.2)	738 (66.0)	
Missing	13 (1.2)	15 (1.3)	
Genotype			
All other genotypes	299 (27.5)	358 (32.0)	.03
HbSS/HbSB^0^	790 (72.5)	760 (68.0)	
Hemoglobin, mean (SD), g/dL	9.23 (1.72)	9.89 (2.03)	<.001
Scores for patient-reported outcome measures, mean (SD)^c^			
Executive difficulties^d^	0.14 (0.98)	0.14 (0.99)	.99
Depression^e^	−0.06 (0.98)	−0.04 (1.03)	.68
Pain^f^	0.06 (0.98)	−0.07 (1.02)	.005
Hydroxyurea use, mean (SD), d/wk	6.53 (2.30)	NA	NA

Abbreviation: NA, not applicable.

SI conversion factor: To convert hemoglobin to grams per liter, multiply by 10.

^a^ *P* values were calculated using χ^2^ test for categorical variables and Wilcoxon rank-sum test for continuous variables.

^b^ The other category comprises any race not otherwise specified.

^c^ Raw item responses were transformed to *z *scores (mean = 0; SD = 1) for analyses; higher scores indicate more problems.

^d^ Measured using 5 self-report items from the Quality of Life in Neurological Disorders Item Bank version 2.0.

^e^ Measured using 4 self-report items from the Patient-Reported Outcomes Measurement Information System Short Form version 1.0 Depression 4a.

^f^ Measured using 2 self-report items from the Adult Sickle Cell Quality of Life Measurement Information System.

### Self-Reported Hydroxyurea Use

**Figure 1.**
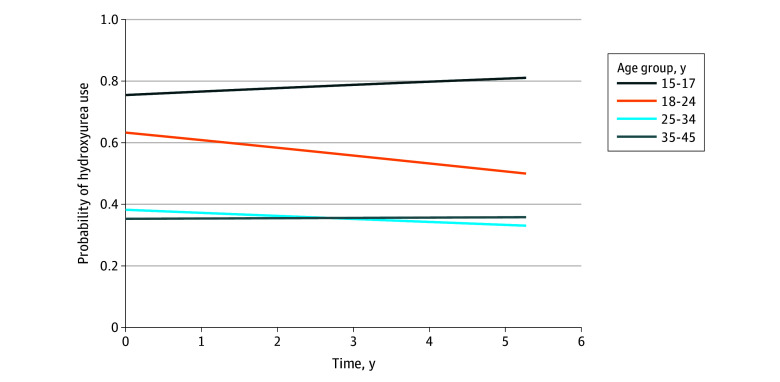
Line Graph of Probability of Hydroxyurea Use Over Time by Age Group (N = 2207) Current hydroxyurea use was measured by self-report (binary) according to the response to the question, “Are you currently taking hydroxyurea?”

Table 2 shows the LME model results for hydroxyurea use, with time included in the analysis. Overall, the rates of hydroxyurea use remained relatively stable during the study period (1089 of 2207 patients [49.3%] at baseline, 887 of 1802 patients [48.7%] at first follow-up, 609 of 1281 patients [47.5%] at second follow-up, and 378 of 783 patients [48.3%] at third follow-up) and were not significantly associated with time. However, patients with HbSS/SB^0^-thalassemia showed declining use (790 of 1550 patients [50.9%] at baseline, 627 of 1276 patients [49.1%] at first follow-up, 429 of 905 patients [47.4%] at second follow-up, and 282 of 586 patients [48.1%] at third follow-up), whereas patients with other genotypes showed increasing (299 of 657 patients [45.5%] at baseline, 250 of 526 patients [47.5%] at first follow-up, 168 of 352 patients [47.7%] at second follow-up, and 90 of 186 patients [48.4%] at third follow-up) use across time points. Compared with patients aged 15 to 17 years at baseline, of whom 109 of 189 (57.7%) reported hydroxyurea use, all older age groups had significantly lower odds of hydroxyurea use (Figure 1), with those aged 18 to 24 years having 61% lower odds of use (OR, 0.39; 95% CI, 0.17-0.92; *P* = .03; 340 of 620 patients [54.8%] reported use), those aged 25 to 34 years having 89% lower odds of use (OR, 0.11; 95% CI, 0.05-0.27; *P* < .001; 410 of 883 patients [46.4%] reported use), and those aged 35 to 45 years having 87% lower odds of use (OR, 0.13; 95% CI, 0.05-0.33; *P* < .001; 227 of 509 patients [44.6%] reported use). Greater symptoms of pain were associated with greater hydroxyurea use where a 1-SD increase in pain measures corresponded to 25% higher odds of use (OR, 1.25; 95% CI, 1.08-1.45; *P* = .002). Higher hemoglobin levels were associated with lower hydroxyurea use, such that 1 g/dL increase in hemoglobin levels corresponded to approximately 53% lower odds of hydroxyurea use (OR, 0.47; 95% CI, 0.42-0.55; *P* < .001). Male patients had more than 6 times the odds of hydroxyurea use than female patients in the longitudinal mixed-effects models (OR, 6.34; 95% CI, 3.88-10.35; *P* < .001). In total, 524 of 942 male patients (55.6%) vs 565 of 1264 female patients (44.7%) reported use at baseline, unadjusted for confounding factors.

**Table 2.** Mixed-Effects Model of Hydroxyurea Use Over Time (N = 2207)^a^

Variable	OR (95% CI)	*P* value
Age group, y		
18-24	0.39 (0.17-0.92)	.03
25-34	0.11 (0.05-0.27)	<.001
35-45	0.13 (0.05-0.33)	<.001
Sex	6.34 (3.88-10.35)	<.001
Education	0.78 (0.45-1.37)	.39
Genotype	0.95 (0.57-1.56)	.83
Hemoglobin, g/dL	0.47 (0.42-0.55)	<.001
Patient-reported outcome measures^b^		
Executive difficulties^c^	1.03 (0.89-1.20)	.65
Depression^d^	0.88 (0.76-1.01)	.08
Pain^e^	1.25 (1.08-1.45)	.002
Time, y	0.98 (0.91-1.07)	.77

Abbreviation: OR, odds ratio.

^a^ Hydroxyurea use was measured by self-report (binary). The reference group for age is 15 to 17 years, the reference group for sex is female, the reference group for education is college graduate or graduate degree, and the reference group for genotype is genotypes other than HbSS/HbSB^0^.

^b^ Raw item responses were transformed to *z* scores (mean = 0, SD = 1) for analyses; higher scores indicate more problems.

^c^ Measured using 5 self-report items from the Quality of Life in Neurological Disorders Item Bank version 2.0.

^d^ Measured using 4 self-report items from the Patient-Reported Outcomes Measurement Information System Short Form version 1.0 Depression 4a.

^e^ Measured with 2 self-report items from the Adult Sickle Cell Quality of Life Measurement Information System.

**Figure 2.**
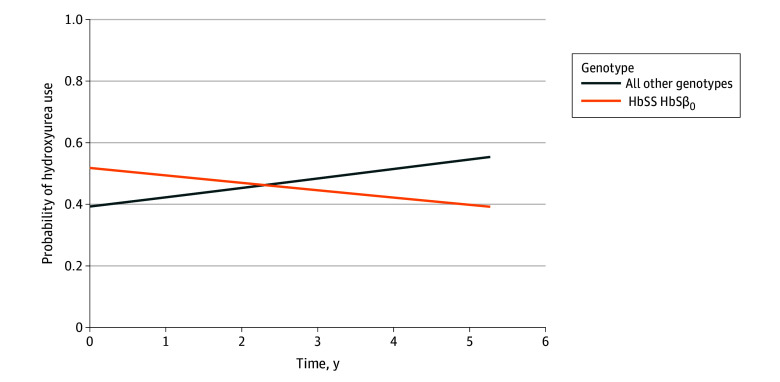
Line Graph of Probability of Hydroxyurea Use Over Time by Genotype (N = 2207) Current hydroxyurea use was measured by self-report (binary) according to the response to the question, “Are you currently taking hydroxyurea?” Genotypes were split into 2 groups: HbSS/HbSB^0^ vs all other genotypes. The time-by-genotype interaction was calculated using a mixed-effects model controlling for age, sex, education, hemoglobin, executive difficulties, depression, pain, and all other time-by-covariate interaction terms (estimate = 0.71; 95% CI, 0.56-0.89; *P* = .004).

After covarying for the demographic and medical characteristics described above, we observed a significant interaction between genotype and time (Figure 2), such that for each additional year since baseline, patients with HbSS/SB^0^-thalassemia had a 24% greater decline in the odds of hydroxyurea use compared with patients with other genotypes (OR, 0.76; 95% CI, 0.63-0.91; *P* = .004), meaning hydroxyurea use decreased more over time for patients with the HbSS/SB^0^-thalassemia genotype. Furthermore, there was an interaction between baseline education and time, such that participants without a college degree had a 32% greater increase in the odds of hydroxyurea use over time compared with participants with a college degree (OR, 1.32; 95% CI, 1.08-1.61; *P* = .007), meaning hydroxyurea use increased more over time for participants without a college degree at baseline. eTable 3 in Supplement 1 shows the mixed-effects model with interaction terms included.

### Self-Reported Hydroxyurea Adherence

Table 3 shows the LME model results for hydroxyurea adherence (number of days taken in the past week) in the group of patients using hydroxyurea. Overall, adherence declined over time (−0.19 days/week/year; 95% CI, −0.24 to −0.14 days/week/year; *P* < .001), showing a gradual downward trend over time. Compared with patients aged 15 to 17 years, patients aged 18 to 24 years and 25 to 34 years reported lower adherence by about half a day per week. Hydroxyurea adherence over time by age group is displayed in eFigure 1 in Supplement 1. Greater self-reported executive difficulties were associated with lower hydroxyurea adherence (−0.17 days/week/year; 95% CI, −0.26 to −0.08 days/week/year; *P* < .001). eTable 4 in Supplement 1 displays the mixed-effects model with interactions included.

**Table 3.** Mixed-Effects Model of Hydroxyurea Adherence Over Time (n = 1089)^a^

Variable	Estimate (95% CI)	*P* value
Age group, y		
18-24	−0.46 (−0.81 to −0.12)	.008
25-34	−0.45 (−0.79 to −0.10)	.01
35-45	−0.2 (−0.58 to 0.18)	.29
Sex	0.09 (−0.10 to 0.29)	.35
Education	−0.05 (−0.30 to 0.19)	.67
Genotype	0.11 (−0.11 to 0.33)	.34
Hemoglobin, g/dL	0.05 (−0.01 to 0.11)	.11
Patient-reported outcome measures^b^		
Executive difficulties^c^	−0.17 (−0.26 to −0.08)	<.001
Depression^d^	−0.02 (−0.11 to 0.07)	.65
Pain^e^	0.00 (−0.09 to 0.09)	.98
Time, y	−0.19 (−0.24 to −0.14)	<.001

^a^ Hydroxyurea adherence was measured by self-report (number of days hydroxyurea taken within the past week). The reference group for age is 15 to 17 years, the reference group for sex is female, the reference group for education is college graduate or graduate degree, and the reference group for genotype is genotypes other than HbSS/HbSB^0^.

^b^ Raw item responses were transformed to *z* scores (mean = 0, SD = 1) for analyses; higher scores indicate more problems.

^c^ Measured using 5 self-report items from the Quality of Life in Neurological Disorders Item Bank version 2.0.

^d^ Measured using 4 self-report items from the Patient-Reported Outcomes Measurement Information System Short Form version 1.0 Depression 4a.

^e^ Measured with 2 self-report items from the Adult Sickle Cell Quality of Life Measurement Information System.

eTable 5 in Supplement 1 displays the stability and cross-lagged effects of executive dysfunction, depression, and pain on hydroxyurea adherence. CLPMs revealed bidirectional temporal associations. Greater executive difficulties were followed by lower adherence; in addition, higher adherence was followed by fewer executive difficulties. Similarly, a bidirectional association for depressive symptoms and adherence was also observed. eFigures 2 and 3 in Supplement 1 display the bidirectional associations between executive difficulties and depression with hydroxyurea adherence, respectively.

## Discussion

To our knowledge, this cohort study is the first to use longitudinal data to identify bidirectional temporal associations between executive difficulties or depression and hydroxyurea adherence among patients with SCD. We identified subgroups of patients who are at greater risk for stopping hydroxyurea treatment, along with modifiable skills to improve hydroxyurea adherence. These findings highlight the need to consider and incorporate cognitive and mental health factors when developing interventions to improve and sustain hydroxyurea adherence in SCD.

Contrary to our hypothesis, we did not observe an overall decline in hydroxyurea use over the course of the study. However, patients with HbSS/SB^0^-thalassemia showed declining use, whereas patients with other genotypes showed increasing use. Patients with HbSS/SB^0^-thalassemia may have stopped treatment in adulthood owing to perceived waning of hydroxyurea’s effectiveness after long-term use.^14^ Patients with other genotypes may initiate hydroxyurea treatment in adulthood because of worsening symptoms. Our findings suggest that there is a great need for pediatricians and pediatric hematologists to initiate hydroxyurea treatment early in life. This practice has the potential to sustain use, as patients may be less likely to initiate hydroxyurea in adulthood, particularly those with HbSS/SB^0^-thalassemia. There was a substantial difference in hydroxyurea use based on sex, with male patients being more likely than female patients to use hydroxyurea (55.6% vs 44.7%). These sex differences are consistent with previous findings^9^ and may be attributable to concerns of teratogenicity of hydroxyurea during pregnancy. Unexpectedly, patients with a college degree at baseline showed a decline over time in hydroxyurea use compared with those without. This may be due, in part, to planned delays for starting a family and pregnancy-related concerns with hydroxyurea use,^39^ as the college-educated group was much more likely to be married, particularly those aged 25 to 34 years.

There was a significant decline in overall self-reported hydroxyurea adherence. This decline extended across genotypes, sexes, and age groups. The broad trajectory of declining adherence over time is consistent with other chronic disease populations, such as statin use in patients with coronary artery disease.^40^ Consistent with previous studies in patients with SCD,^9^ young adults (aged 18-24 years) had the lowest levels of hydroxyurea adherence across age groups. This decline in adherence coincides with a significant transition period in health care as well as changes in academics, occupation, familial relationships, and emotional functioning.^41^

Executive functioning deficits and depressive symptoms are common complications of SCD that tend to become more prevalent as patients age and are known to be associated with environmental factors (eg, poverty).^42,43^ Previous cross-sectional studies have highlighted associations among these complications and hydroxyurea adherence,^20,29^ but it was unknown whether these complications resulted from or contributed to poor adherence. We observed that executive difficulties and depressive symptoms were associated with lower future adherence, and that lower adherence was associated with greater future executive difficulties and depressive symptoms. These bidirectional temporal associations highlight that early treatment with hydroxyurea may prevent disease-related mental health complications, and that preserved mental health may serve as a protective factor to promote adherence as patients age.

Across study time points, greater self-reported pain was positively associated with hydroxyurea use. Practitioners may be more inclined to prescribe hydroxyurea to alleviate symptoms. Furthermore, patients may be more motivated to use hydroxyurea to treat their pain. However, among patients taking hydroxyurea, self-reported pain was not associated with adherence. This suggests that perceived pain was not a motivating factor for patients to sustain adherence and that pain symptoms did not interfere with adherence. Although pain was not directly associated with adherence, pain may have an indirect effect on adherence through impacts on mental health and cognitive functioning.^30^ In previous adherence interventions among patients with SCD, greater pain was associated with less intervention engagement^44^ and worse intervention efficacy.^45^ Pain may initially prompt the practitioner and/or patient to use hydroxyurea and may interfere with intervention engagement. However, our data suggest that pain does not directly influence adherence.

Interventions to promote hydroxyurea adherence have primarily focused on providing education, increasing self-efficacy, and improving self-monitoring. Some of these interventions have incorporated the use of technology (eg, text-message reminders or health-related smartphone applications),^45,46,47^ and others have relied on familial or health-system support.^48^ Broadly, interventions that have targeted self-efficacy and self-monitoring at the individual level have resulted in adherence improvements.^49^ Our findings support the importance of addressing self-monitoring behaviors as they relate to disruptions in executive functioning skills. Although mood and depressive symptoms were not explicitly addressed in previous interventions, depression is closely linked to an individual’s self-efficacy^50,51^ and is known to respond to interventions targeting self-efficacy.^52^ Interventions that use cognitive behavioral therapy strategies (eg, challenging and reframing unhelpful thinking patterns or behavioral activation) are likely to positively influence both the cognitive and mental health factors contributing to medication nonadherence.^52^ Furthermore, our findings highlight the importance of integrating cognitive and mental health screening into adherence interventions and routine care for patients with SCD.

### Limitations

Strengths of the study include a longitudinal design spanning several years, a large sample size, and data collection at 8 sites across the US. However, several study limitations exist. Traditional CLPMs do not separate stable between-person differences from within-person changes. This means that the observed cross-lagged associations, such as executive difficulties and adherence, may come from differences between individuals rather than within-person changes over time.^53^ Most study outcomes were based on self-reported patient data. This type of measurement is prone to substantial biases, particularly on measures of use and adherence, where patients tend to report greater use and adherence than is observed through more objective measures. The use of self-reported hydroxyurea adherence enabled detection of within-person temporal variation, a key feature necessary for CLPM. Because adherence behavior fluctuates with daily cognitive load, mood, and contextual stressors, self-report provided a proximal indicator of these dynamic processes. Future research integrating biological adherence markers with real-time self-report measures like ecological momentary assessment may better elucidate the causal mechanisms linking neurocognitive function, mood, and sustained hydroxyurea use. Future studies should examine self-reported barriers and explore systems-level factors impacting hydroxyurea use and adherence.

## Conclusions

In this cohort study of patients with SCD, we identified previously unknown trends in hydroxyurea continuation and user-level factors associated with adherence. Given few alternative treatment options, declining use of hydroxyurea among patients with HbSS/SB^0^-thalassemia genotypes raises important concerns about ongoing disease management for these patients. For patients taking hydroxyurea, interventions targeting self-efficacy and self-monitoring are likely to promote sustained adherence by mitigating the negative effects of executive difficulties and depression. Future studies should test behavioral interventions targeting cognitive and affective mechanisms of adherence.

## Notes

Supplement 1. eTable 1. Patient characteristics at baseline and across timepoints completed eTable 2. Patient characteristics at baseline and across timepoints completed for the group taking hydroxyurea eTable 3. Mixed-effects model of hydroxyurea usage over time with all time interaction terms (n = 2,207) eTable 4. Mixed-effects model of hydroxyurea adherence over time with all time interaction terms (n = 1,089) eTable 5. Cross-lagged models examining associations between executive difficulties, depression, or pain and hydroxyurea adherence (n = 1,089) eFigure 1. Hydroxyurea adherence over time by age group (n = 1,089). Hydroxyurea adherence was measured by self-report (number of days hydroxyurea taken within the past week) eFigure 2. Cross-lagged models of hydroxyurea adherence and executive difficulties (n = 1,089) eFigure 3. Cross-lagged models of hydroxyurea adherence and depression (n = 1,089)

Supplement 2. Data Sharing Statement
