# Supplementary material for: Sustainment of Hydroxyurea Adherence in Patients With Sickle Cell Disease
Source: JAMA Netw Open. 2026 May 6;9(5):e2611257. doi: 10.1001/jamanetworkopen.2026.11257 (PMC13150639; doi:10.1001/jamanetworkopen.2026.11257)
Supplement: Supplement 1. — eTable 1. Patient characteristics at baseline and across timepoints completed eTable 2. Patient characteristics at baseline and across timepoints completed for the group taking hydroxyurea eTable 3. Mixed-effects model of hydroxyurea usage over time with all time interaction terms (n = 2,207) eTable 4. Mixed-effects model of hydroxyurea adherence over time with all time interaction terms (n = 1,089) eTable 5. Cross-lagged models examining associations between executive difficulties, depression, or pain and hydroxyurea adherence (n = 1,089) eFigure 1. Hydroxyurea adherence over time by age group (n = 1,089). Hydroxyurea adherence was measured by self-report (number of days hydroxyurea taken within the past week) eFigure 2. Cross-lagged models of hydroxyurea adherence and executive difficulties (n = 1,089) eFigure 3. Cross-lagged models of hydroxyurea adherence and depression (n = 1,089) [file jamanetwopen-e2611257-s001.pdf]

Supplemental Online Content

Heitzer AM, Wooten Z, Luo G, et al; Sickle Cell Disease Implementation Consortium. Sustainment of hydroxyurea adherence in patients with sickle cell disease. *JAMA Netw Open*. 2026;9(5):e2611257. doi:10.1001/jamanetworkopen.2026.11257

eTable 1. Patient characteristics at baseline and across timepoints completed

eTable 2. Patient characteristics at baseline and across timepoints completed for the group taking hydroxyurea

eTable 3. Mixed-effects model of hydroxyurea usage over time with all time interaction terms (n = 2,207)

eTable 4. Mixed-effects model of hydroxyurea adherence over time with all time interaction terms (n = 1,089)

eTable 5. Cross-lagged models examining associations between executive difficulties, depression, or pain and hydroxyurea adherence (n = 1,089)

eFigure 1. Hydroxyurea adherence over time by age group (n = 1,089). Hydroxyurea adherence was measured by self-report (number of days hydroxyurea taken within the past week)

eFigure 2. Cross-lagged models of hydroxyurea adherence and executive difficulties (n = 1,089)

eFigure 3. Cross-lagged models of hydroxyurea adherence and depression (n = 1,089)

This supplemental material has been provided by the authors to give readers additional information about their work.

© 2026 Heitzer AM et al. *JAMA Network Open*.

eTable 1. Patient characteristics at baseline and across timepoints completed.

Variable	Baseline (*N* = 2,207)	Timepoint 1 (*N* = 1,802)	Timepoint 2 (*n* = 1,281)	Timepoint 3 (*n* = 783)
	n (%)	*n* (%)	*n* (%)	*n* (%)
Sex				
Female	1265 (57.3%)	1055 (58.5%)	771 (60.2%)	485 (61.9%)
Male	942 (42.7%)	747 (41.5%)	510 (39.8%)	298 (38.1%)
Race				
Asian	4 (0.2%)	4 (0.2%)	3 (0.2%)	2 (0.3%)
Black	2164 (98.1%)	1763 (97.8%)	1255 (98.0%)	776 (99.1%)
Native American	3 (0.1%)	3 (0.2%)	2 (0.2%)	0 (0%)
Other	28 (1.3%)	27 (1.5%)	18 (1.4%)	4 (0.5%)
White	8 (0.4%)	5 (0.3%)	3 (0.2%)	1 (0.1%)
Age Group				
15 to 17	189 (8.6%)	145 (8.0%)	128 (10.0%)	82 (10.5%)
18 to 24	620 (28.1%)	499 (27.7%)	331 (25.8%)	208 (26.6%)
25 to 34	883 (40.0%)	720 (40.0%)	517 (40.4%)	319 (40.7%)
35 to 45	509 (23.1%)	434 (24.1%)	305 (23.8%)	174 (22.2%)
Unknown	6 (0.3%)	4 (0.2%)	0 (0.0%)	0 (0.0%)
Income				
$25,000 or less	1044 (47.3%)	831 (46.1%)	588 (45.9%)	361 (46.1%)
$25,001–$50,000	440 (19.9%)	370 (20.5%)	262 (20.5%)	159 (20.3%)
$50,001–$75,000	212 (9.6%)	183 (10.2%)	132 (10.3%)	86 (11.0%)
$75,001–$100,000	113 (5.1%)	98 (5.4%)	72 (5.6%)	45 (5.7%)
$100,001+	144 (6.5%)	125 (6.9%)	99 (7.7%)	62 (7.9%)
Unknown	254 (11.5%)	195 (10.8%)	128 (10.0%)	70 (8.9%)
Education				
Less than high school	60 (2.7%)	49 (2.7%)	40 (3.1%)	17 (2.2%)
Some high school	304 (13.8%)	235 (13.0%)	183 (14.3%)	114 (14.6%)
High school graduate/GED	605 (27.4%)	484 (26.9%)	331 (25.8%)	198 (25.3%)
Some college/vocational training	702 (31.8%)	578 (32.1%)	400 (31.2%)	246 (31.4%)
College graduate	311 (14.1%)	265 (14.7%)	189 (14.8%)	113 (14.4%)
Some graduate/professional school	42 (1.9%)	33 (1.8%)	25 (2.0%)	20 (2.6%)
Graduate/professional degree	151 (6.8%)	133 (7.4%)	100 (7.8%)	63 (8.0%)
Missing	32 (1.5%)	25 (1.4%)	13 (1.0%)	12 (1.5%)
Marital status				
Not applicable (child)	241 (10.9%)	183 (10.2%)	145 (11.3%)	90 (11.5%)
Married	233 (10.6%)	203 (11.3%)	132 (10.3%)	86 (11.0%)
Living as married	76 (3.4%)	67 (3.7%)	43 (3.4%)	31 (4.0%)

© 2026 Heitzer AM et al. *JAMA Network Open*.

Divorced/separated	126 (5.7%)	101 (5.6%)	73 (5.7%)	46 (5.9%)
Widowed	0 (0%)	0 (0%)	0 (0%)	0 (0%)
Never married	1503 (68.1%)	1228 (68.1%)	878 (68.5%)	524 (66.9%)
Missing	28 (1.3%)	20 (1.1%)	10 (0.8%)	6 (0.8%)
**Genotype**				
All other genotypes	657 (29.8%)	526 (29.2%)	352 (27.5%)	186 (23.8%)
HbSS/HbSB^0	1550 (70.2%)	1276 (70.8%)	905 (70.6%)	586 (74.8%)
Missing	0 (0.0%)	0 (0.0%)	24 (1.9%)	11 (1.4%)
	Mean (SD)	Mean (SD)	Mean (SD)	Mean (SD)
Hemoglobin (g/dL)	9.56 (1.91)	9.55 (1.90)	9.54 (1.88)	9.59 (1.82)
[a] Executive difficulties*	0.14 (0.987)	0.13 (0.977)	0.14 (0.97)	0.13 (0.97)
[b] Depression*	-0.05 (1.01)	-0.06 (0.99)	-0.04 (1.00)	-0.06 (0.96)
[c] Pain*	-0.01 (1.00)	-0.016 (0.99)	-0.04 (0.98)	-0.02 (0.97)

*Raw item responses were transformed to *z*-scores (mean = 0, SD = 1) for analyses.
[a] Measured using 5 self-report items from the Neuro-QoL Item Bank v 2.0.
[b] Measured using 4 self-report items from the PROMIS Short Form v1.0 Depression 4a.
[c] Measured with 2 self-report items item from the ASCQ-ME.
Abbreviations: GED, general educational development; SD, standard deviation.

© 2026 Heitzer AM et al. *JAMA Network Open*.

eTable 2. Patient characteristics at baseline and across timepoints completed for the group taking hydroxyurea.

	Baseline (*n* = 1,089) *n* (%)	Timepoint 1 (*n* = 877) *n* (%)	Timepoint 2 (*n* = 609) *n* (%)	Timepoint 3 (*n* = 378) *n* (%)
Sex				
Female	565 (51.9%)	470 (53.6%)	345 (56.7%)	211 (55.8%)
Male	524 (48.1%)	407 (46.4%)	264 (43.3%)	167 (44.2%)
Race				
Asian	1 (0.1%)	1 (0.1%)	1 (0.2%)	1 (0.3%)
Black	1066 (97.9%)	854 (97.4%)	593 (97.4%)	375 (99.2%)
Native American	0 (0%)	1 (0.1%)	1 (0.2%)	0 (0%)
Other	17 (1.6%)	17 (1.9%)	11 (1.8%)	1 (0.3%)
White	5 (0.5%)	4 (0.5%)	3 (0.5%)	1 (0.3%)
Age Group				
15 to 17	109 (10.0%)	89 (10.1%)	74 (12.2%)	48 (12.7%)
18 to 24	340 (31.2%)	264 (30.1%)	169 (27.8%)	105 (27.8%)
25 to 34	410 (37.6%)	326 (37.2%)	223 (36.6%)	149 (39.4%)
35 to 45	227 (20.8%)	195 (22.2%)	143 (23.5%)	76 (20.1%)
Unknown	3 (0.3%)	3 (0.3%)	0 (0%)	0 (0%)
Income				
$25,000 or less	512 (47.0%)	403 (46.0%)	280 (46.0%)	176 (46.6%)
$25,001–$50,000	208 (19.1%)	177 (20.2%)	118 (19.4%)	69 (18.3%)
$50,001–$75,000	102 (9.4%)	90 (10.3%)	65 (10.7%)	42 (11.1%)
$75,001–$100,000	60 (5.5%)	50 (5.7%)	37 (6.1%)	25 (6.6%)
$100,001+	73 (6.7%)	55 (6.3%)	44 (7.2%)	30 (7.9%)
Unknown	134 (12.3%)	102 (11.6%)	65 (10.7%)	36 (9.5%)
Education				
Less than high school	34 (3.1%)	29 (3.3%)	22 (3.6%)	12 (3.2%)
Some high school	154 (14.1%)	128 (14.6%)	100 (16.4%)	65 (17.2%)
High school graduate/GED	297 (27.3%)	246 (28.1%)	162 (26.6%)	106 (28.0%)
Some college/vocational training	336 (30.9%)	269 (30.7%)	180 (29.6%)	107 (28.3%)
College graduate	156 (14.3%)	115 (13.1%)	83 (13.6%)	48 (12.7%)
Some graduate/professional school	21 (1.9%)	15 (1.7%)	9 (1.5%)	6 (1.6%)
Graduate/professional degree	72 (6.6%)	58 (6.6%)	45 (7.4%)	27 (7.1%)
Missing	19 (1.7%)	17 (1.9%)	8 (1.3%)	7 (1.9%)
Marital Status				
Not applicable (child)	132 (12.1%)	107 (12.2%)	76 (12.5%)	52 (13.8%)
Married	104 (9.6%)	90 (10.3%)	61 (10.0%)	39 (10.3%)
Living as married	23 (2.1%)	24 (2.7%)	17 (2.8%)	17 (4.5%)
Divorced/separated	52 (4.8%)	36 (4.1%)	31 (5.1%)	14 (3.7%)

© 2026 Heitzer AM et al. *JAMA Network Open*.

Widowed	0 (0%)	0 (0%)	0 (0%)	0 (0%)
Never married	765 (70.2%)	612 (69.8%)	419 (68.8%)	253 (66.9%)
Missing	13 (1.2%)	8 (0.9%)	5 (0.8%)	3 (0.8%)
**Genotype**				
All other genotypes	299 (27.5%)	250 (28.5%)	168 (27.6%)	90 (23.8%)
HbSS/HbSB^0	790 (72.5%)	627 (71.5%)	429 (70.4%)	282 (74.6%)
Missing	0 (0.0%)	0 (0.0%)	12 (2.0%)	6 (1.6%)
	Mean (SD)	Mean (SD)	Mean (SD)	Mean (SD)
Hemoglobin (g/dL)	9.23 (1.73)	9.25 (1.71)	9.28 (1.70)	9.25 (1.66)
[a] Executive functioning*	0.14 (0.97)	0.15 (0.98)	0.14 (0.97)	0.13 (0.97)
[b] Depression*	-0.05 (0.98)	-0.07 (0.97)	-0.09 (0.95)	-0.09 (0.90)
[c] Pain*	0.05 (0.98)	0.04 (0.97)	0.031 (0.97)	0.01 (0.96)
Hydroxyurea use (days/week)	6.53 (2.30)	6.57 (2.30)	6.58 (2.29)	6.72 (2.23)

*Raw item responses were transformed to *z*-scores (mean = 0, SD = 1) for analyses.
[a] Measured using 5 self-report items from the Neuro-QoL Item Bank v 2.0.
[b] Measured using 4 self-report items from the PROMIS Short Form v1.0 Depression 4a.
[c] Measured with 2 self-report items item from the ASCQ-ME.
Abbreviations: GED, general educational development; SD, standard deviation.

© 2026 Heitzer AM et al. *JAMA Network Open*.

eTable 3. Mixed-effects model of hydroxyurea usage over time with all time interaction terms (*n* = 2,207).

Term	Odds ratio	Lower CI	Upper CI	P
Time (years)	1.10	0.76	1.60	0.60
18 to 24 years	0.38	0.16	0.91	0.03
25 to 34 years	0.11	0.04	0.26	<0.001
35 to 45 years	0.12	0.05	0.32	<0.001
Sex	6.51	3.95	10.71	<0.001
Education	0.80	0.45	1.40	0.44
Genotype	0.91	0.55	1.51	0.71
Hemoglobin (g/dL)	0.24	0.18	0.31	<0.001
[a] Executive difficulties[#]	1.03	0.89	1.20	0.68
[b] Depression[#]	0.88	0.76	1.02	0.09
[c] Pain[#]	1.27	1.09	1.47	0.002
Time (years) * 18 to 24 years	0.86	0.64	1.16	0.33
Time (years) * 25 to 34 years	0.94	0.69	1.26	0.66
Time (years) * 35 to 45 years	0.94	0.68	1.32	0.74
Time (years) * sex	0.91	0.77	1.08	0.27
Time (years) * education	1.32	1.08	1.61	0.007
Time (years) * genotype	0.76	0.63	0.91	0.004
Time (years) * hemoglobin	0.97	0.89	1.06	0.47
Time (years) * executive difficulties	0.98	0.90	1.07	0.67
Time (years) * depression	1.00	0.91	1.10	0.98
Time (years) * pain	0.95	0.87	1.05	0.31

Hydroxyurea usage was measured via self-report (binary); reference group for age = 15 to 17 years; reference group for sex = female sex; reference group for education = college graduate or graduate degree; reference group for genotype = genotypes other than HbSS/HbSB^0.

[#]Raw item responses were transformed to *z*-scores (mean = 0, SD = 1) for analyses; higher scores indicate more problems.

[a] Measured using 5 self-report items from the Neuro-QoL Item Bank v 2.0.

[b] Measured using 4 self-report items from the PROMIS Short Form v1.0 Depression 4a.

[c] Measured with 2 self-report items item from the ASCQ-ME.

Abbreviations: CI, confidence interval.

© 2026 Heitzer AM et al. *JAMA Network Open*.

eTable 4. Mixed-effects model of hydroxyurea adherence over time with all time interaction terms (*n* = 1,089).

Term	Estimate	Lower CI	Upper CI	P
Time (years)	-0.18	-0.37	0.02	0.08
18 to 24 years	-0.48	-0.82	-0.13	0.007
25 to 34 years	-0.46	-0.81	-0.11	0.010
35 to 45 years	-0.21	-0.59	0.17	0.27
Sex	0.09	-0.10	0.29	0.35
Education	-0.05	-0.30	0.20	0.69
Genotype	0.11	-0.11	0.33	0.33
Hemoglobin (g/dL)	0.04	-0.01	0.10	0.13
[a] Executive difficulties[#]	-0.18	-0.27	-0.09	<0.001
[b] Depression[#]	-0.01	-0.11	0.08	0.77
[c] Pain[#]	0.00	-0.09	0.09	0.94
Time (years) * 18 to 24 years	0.06	-0.13	0.24	0.54
Time (years) * 25 to 34 years	0.03	-0.15	0.22	0.73
Time (years) * 35 to 45 years	0.07	-0.14	0.28	0.52
Time (years) * sex	-0.02	-0.14	0.09	0.67
Time (years) * education	0.02	-0.13	0.15	0.86
Time (years) * genotype	-0.02	-0.14	0.11	0.84
Time (years) * hemoglobin	-0.01	-0.07	0.06	0.78
Time (years) * executive difficulties	0.03	-0.03	0.09	0.33
Time (years) * depression	-0.06	-0.13	0.00	0.06
Time (years) * pain	0.03	-0.02	0.10	0.30

Hydroxyurea usage was measured via self-report (binary); reference group for age = 15 to 17 years; reference group for sex = female sex; reference group for education = college graduate or graduate degree; reference group for genotype = genotypes other than HbSS/$HbSB^0$.

[#]Raw item responses were transformed to *z*-scores (mean = 0, SD = 1) for analyses; higher scores indicate more problems.

[a] Measured using 5 self-report items from the Neuro-QoL Item Bank v 2.0.

[b] Measured using 4 self-report items from the PROMIS Short Form v1.0 Depression 4a.

[c] Measured with 2 self-report items from the ASCQ-ME.

Abbreviations: CI, confidence interval.

© 2026 Heitzer AM et al. *JAMA Network Open*.

eTable 5. Cross-lagged models examining associations between executive difficulties, depression, or pain and hydroxyurea adherence (*n* = 1,089).

[a] Executive difficulties*

Response	Predictor	Estimate	SE	Path type	p
HU days 2	HU days 1	0.290	0.045	Stability (HU days)	0.000
HU days 3	HU days 2	0.395	0.053	Stability (HU days)	0.000
HU days 4	HU days 3	0.573	0.073	Stability (HU days)	0.000
Executive 2	Executive 1	0.538	0.034	Stability (Executive)	0.000
Executive 3	Executive 2	0.659	0.041	Stability (Executive)	0.000
Executive 4	Executive 3	0.581	0.053	Stability (Executive)	0.000
HU days 2	Executive 1	-0.016	0.007	**Cross-lagged: Executive ➔ HU days**	**0.034**
HU days 3	Executive 2	-0.002	0.008	Cross-lagged: Executive ➔ HU days	0.76
HU days 4	Executive 3	-0.020	0.008	**Cross-lagged: Executive ➔ HU days**	**0.012**
Executive 2	HU days 1	-0.179	0.137	Cross-lagged: HU days ➔ Executive	0.19
Executive 3	HU days 2	-0.424	0.190	**Cross-lagged: HU days ➔ Executive**	**0.026**
Executive 4	HU days 3	-0.170	0.280	Cross-lagged: HU days ➔ Executive	0.55
HU days 1	Age	0.554	0.187	Other	0.003
HU days 1	Sex	0.120	0.139	Other	0.39
HU days 1	Genotype	0.141	0.160	Other	0.38
Executive 1	Age	0.726	0.982	Other	0.46
Executive 1	Sex	-1.736	0.576	Other	0.003
Executive 1	Genotype	0.206	0.639	Other	0.75

[b] Depression*

Response	Predictor	Estimate	SE	Path type	p
HU days 2	HU days 1	0.296	0.044	Stability (HU days)	0.000
HU days 3	HU days 2	0.385	0.053	Stability (HU days)	0.000
HU days 4	HU days 3	0.578	0.074	Stability (HU days)	0.000
Depression 2	Depression 1	0.499	0.038	Stability (Depression)	0.000
Depression 3	Depression 2	0.575	0.043	Stability (Depression)	0.000
Depression 4	Depression 3	0.587	0.053	Stability (Depression)	0.000
HU days 2	Depression 1	-0.008	0.007	Cross-lagged: Depression ➔ HU days	0.23
HU days 3	Depression 2	-0.015	0.007	**Cross-lagged: Depression ➔ HU days**	**0.045**
HU days 4	Depression 3	-0.014	0.009	Cross-lagged: Depression ➔ HU days	0.11
Depression 2	HU days 1	-0.382	0.156	**Cross-lagged: HU days ➔ Depression**	**0.01**
Depression 3	HU days 2	-0.080	0.212	Cross-lagged: HU days ➔ Depression	0.71
Depression 4	HU days 3	0.191	0.326	Cross-lagged: HU days ➔ Depression	0.56
HU days 1	Age	0.539	0.187	Other	0.004
HU days 1	Sex	0.119	0.138	Other	0.39
HU days 1	Genotype	0.133	0.160	Other	0.41
Depression 1	Age	-4.706	0.669	Other	0.000
Depression 1	Sex	-1.698	0.579	Other	0.003
Depression 1	Genotype	-0.809	0.692	Other	0.24

[c] Pain*

© 2026 Heitzer AM et al. *JAMA Network Open*.

Response	Predictor	Estimate	SE	Path type	p
HU days 2	HU days 1	0.300	0.044	Stability (HU days)	0.000
HU days 3	HU days 2	0.396	0.053	Stability (HU days)	0.000
HU days 4	HU days 3	0.582	0.074	Stability (HU days)	0.000
Pain 2	Pain 1	0.530	0.031	Stability (Pain)	0.000
Pain 3	Pain 2	0.594	0.034	Stability (Pain)	0.000
Pain 4	Pain 3	0.584	0.045	Stability (Pain)	0.000
HU days 2	Pain 1	0.004	0.007	Cross-lagged: Pain ➔ HU days	0.54
HU days 3	Pain 2	-0.001	0.007	Cross-lagged: Pain ➔ HU days	0.87
HU days 4	Pain 3	-0.009	0.009	Cross-lagged: Pain ➔ HU days	0.35
Pain 2	HU days 1	-0.246	0.153	Cross-lagged: HU days ➔ Pain	0.11
Pain 3	HU days 2	-0.127	0.181	Cross-lagged: HU days ➔ Pain	0.48
Pain 4	HU days 3	0.170	0.271	Cross-lagged: HU days ➔ Pain	0.53
HU days 1	Age	0.553	0.187	Other	0.003
HU days 1	Sex	0.125	0.139	Other	0.37
HU days 1	Genotype	0.136	0.160	Other	0.39
Pain 1	Age	-5.616	0.906	Other	0.000
Pain 1	Sex	-1.274	0.575	Other	0.02
Pain 1	Genotype	-0.593	0.653	Other	0.36

Hydroxyurea adherence was measured via self-report (number of days used per week); reference group for age = 18 and over; reference group for sex = female sex; reference group for education = college graduate or graduate degree; reference group for genotype = genotypes other than HbSS/HbSB^0

[#]Raw item responses were transformed to *z*-scores (mean = 0, SD = 1) for analyses; higher scores indicate more problems.

[a] Measured using 5 self-report items from the Neuro-QoL Item Bank v 2.0.

[b] Measured using 4 self-report items from the PROMIS Short Form v1.0 Depression 4a.

[c] Measured with 2 self-report items item from the ASCQ-ME.

Abbreviations: HU, hydroxyurea; SE, standard error.

© 2026 Heitzer AM et al. *JAMA Network Open*.

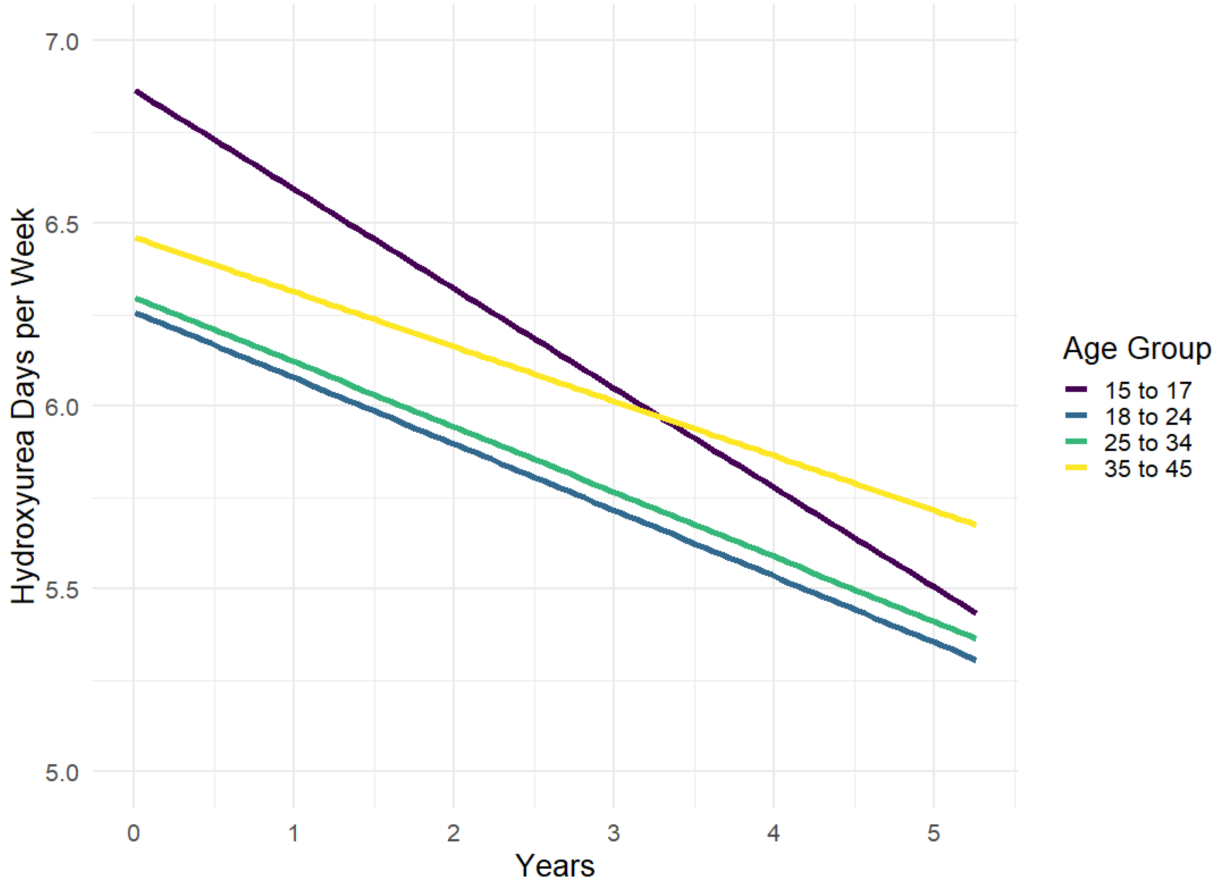

eFigure 1. Hydroxyurea adherence over time by age group (*n* = 1,089). Hydroxyurea adherence was measured by self-report (number of days hydroxyurea taken within the past week).

© 2026 Heitzer AM et al. *JAMA Network Open*.

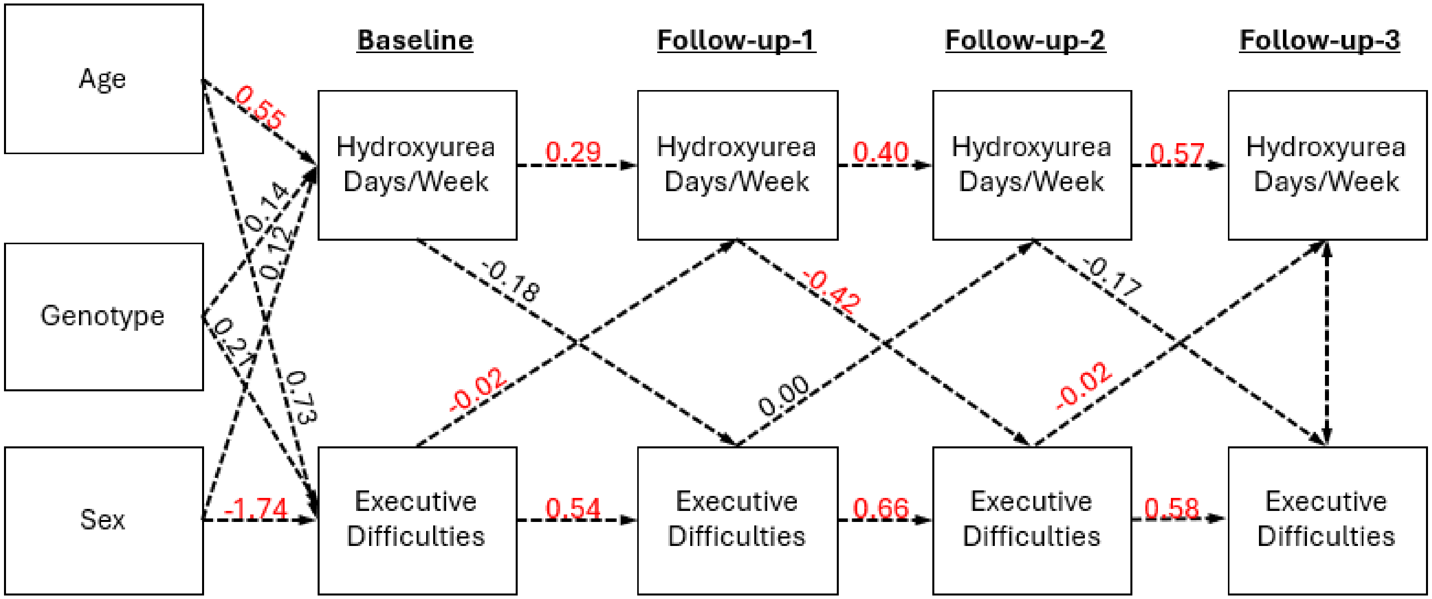

eFigure 2. Cross-lagged models of hydroxyurea adherence and executive difficulties (*n* = 1,089). Associations in red are statistically significant (P < 0.05). Hydroxyurea adherence was measured by self-report (number of days hydroxyurea taken within the past week). Executive difficulties were measured using 5 self-report items from the Neuro-QoL Item Bank v 2.0. Raw item responses from the Neuro-QoL were transformed to *z*-scores (mean = 0, standard deviation = 1) for analyses. Higher executive difficulties scores indicate more problems.

© 2026 Heitzer AM et al. *JAMA Network Open*.

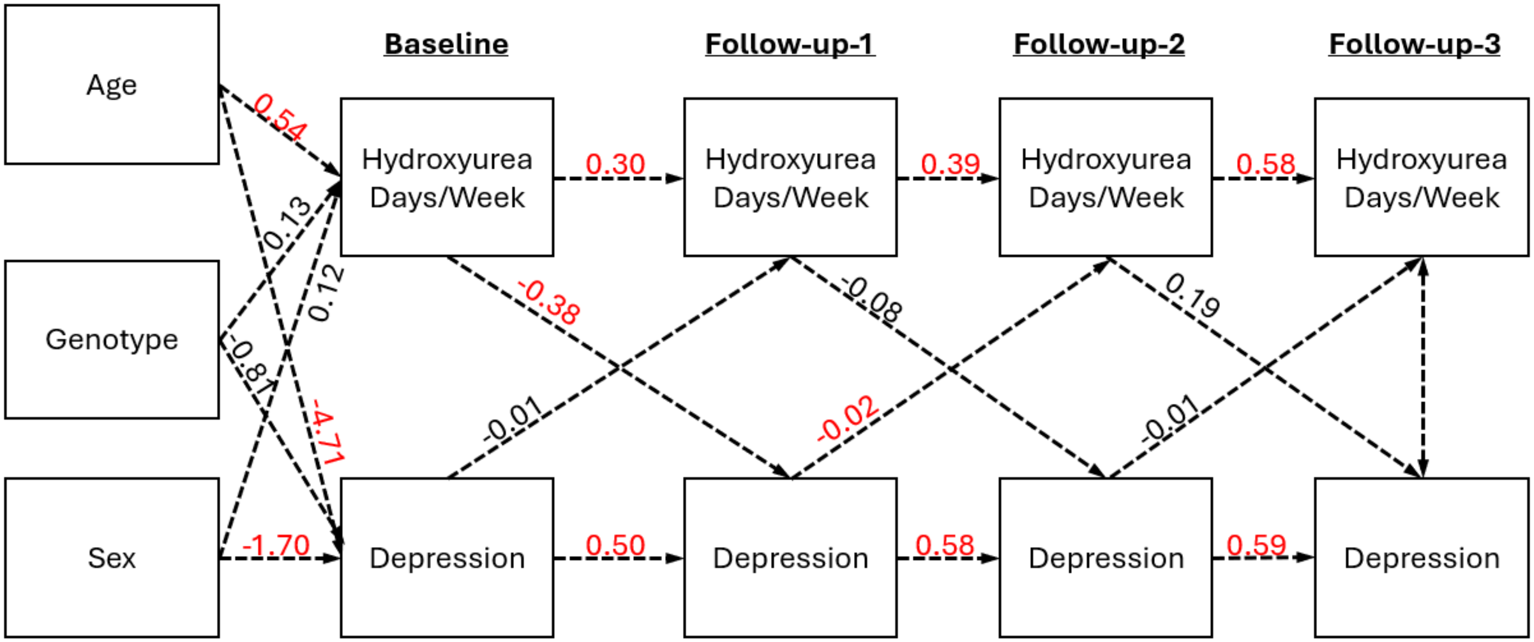

eFigure 3. Cross-lagged models of hydroxyurea adherence and depression (*n* = 1,089). Associations in red are statistically significant (P < 0.05). Hydroxyurea adherence was measured by self-report (number of days hydroxyurea taken within the past week). Depression was measured using 4 self-report items from the PROMIS Short Form v1.0 Depression 4a. Raw item responses from the PROMIS were transformed to *z*-scores (mean = 0, standard deviation = 1) for analyses. Higher depression scores indicate more problems.

© 2026 Heitzer AM et al. *JAMA Network Open*.
