# Supplementary material for: Sustainment of Hydroxyurea Adherence in Patients With Sickle Cell Disease
Source: JAMA Netw Open. 2026 May 6;9(5):e2611257. doi: 10.1001/jamanetworkopen.2026.11257 (PMC13150639; doi:10.1001/jamanetworkopen.2026.11257)
Supplement: Supplement 2. — Data Sharing Statement [file jamanetwopen-e2611257-s002.pdf]

Data Sharing Statement

Heitzer. Sustainment of Hydroxyurea Adherence in Patients With Sickle Cell Disease. *JAMA Netw Open*. Published May 06, 2026. doi:10.1001/jamanetworkopen.2026.11257

Data
Data available: Yes
Data types: Deidentified participant data
How to access data: Data are publicly available from the National Heart, Lung, and Blood Institute Data Repository at https://biolincc.nhlbi.nih.gov/studies/scdic_registry/
When available: With publication

Supporting Documents
Document types: None

Additional Information
Who can access the data: Anyone requesting the data.
Types of analyses: The data are publicly available to anyone.
Mechanisms of data availability: Data are publicly available from the National Heart, Lung, and Blood Institute Data Repository at https://biolincc.nhlbi.nih.gov/studies/scdic_registry/
